# Ampicillin Depletion and Withdrawal Period in Broilers: Tissue Residue Analysis After Intramuscular Administration

**DOI:** 10.3390/ani16121821

**Published:** 2026-06-12

**Authors:** Paula Cortés, Maximiliano Castillo, Katherine Codoceo Valenzuela, Kevin Manríquez González, Belén Pinto, Ekaterina Pokrant, Aldo Maddaleno, Sebastián Zavala, Andrés Flores, Javiera Cornejo

**Affiliations:** 1 Laboratory of Veterinary Pharmacology (FARMAVET), Faculty of Veterinary and Animal Sciences, University of Chile, Santa Rosa 11735, La Pintana, Santiago 8820808, Chile; paula.cortes@ug.uchile.cl (P.C.); m.castillo.1@ug.uchile.cl (M.C.); belen.pinto@ug.uchile.cl (B.P.); amaddaleno@veterinaria.uchile.cl (A.M.); sebastian.zavala@uchile.cl (S.Z.); andres.flores@veterinaria.uchile.cl (A.F.); 2 Programa de Doctorado en Ciencias Silvoagropecuarias y Veterinarias, Campus Sur Universidad de Chile, Santa Rosa 11315, La Pintana, Santiago 8820808, Chile; 3 Department of Preventive Animal Medicine, Faculty of Veterinary and Animal Sciences, University of Chile, Santa Rosa 11735, La Pintana, Santiago 8820808, Chile; katherine.codoceo@ug.uchile.cl (K.C.V.); kevin.manriquez@ug.uchile.cl (K.M.G.); 4 Facultad de Medicina Veterinaria, Universidad San Sebastián, Bellavista 7, Recoleta, Santiago 8420524, Chile; katiavalerievna@uchile.cl; 5 Laboratory of Food Safety (INOCUIVET), Department of Preventive Animal Medicine, Faculty of Veterinary and Animal Sciences, University of Chile, Santa Rosa 11735, La Pintana, Santiago 8820808, Chile

**Keywords:** ampicillin residue, depletion, withdrawal period, marker residue, edible tissues, broiler chicken, confirmatory method, LC-MS/MS

## Abstract

Ampicillin residues in animal-derived foods may cause allergic reactions and promote antimicrobial resistance in consumers; however, data on residue behavior in poultry remain limited. This study aimed to evaluate the depletion of ampicillin in muscle and skin plus fat of broiler chickens. Thirty birds were treated with ampicillin intramuscularly (20 mg kg^−1^ every 24 h for three days) and sacrificed at 0.5, 1, 2, 5, and 9 days post-administration. Samples were analyzed by liquid chromatography coupled with tandem mass spectrometry, a method successfully validated according to Commission Implementing Regulation (EU) 2021/808, VICH GL49 and GL2. Quantification was performed by linear regression from matrix-matched calibration curves. Residue depletion was evaluated following the European Medicines Agency guidelines. Ampicillin residues in muscle were detected only during the first 24 h post-administration (6.50–8.48 µg kg^−1^). Residues in skin plus fat remained detectable until day 5 post-administration (6.87–59.88 µg kg^−1^). Based on this, the withdrawal period calculated for skin plus fat was 9 days considering EU maximum residue limit (MRL) and 19 days considering method limit of quantification, with 95% confidence. These results provide critical data on ampicillin residue kinetics under controlled experimental conditions, supporting risk assessments and the establishment of MRLs in broiler chickens by the Codex Alimentarius.

## Simple Summary

Ampicillin is used to treat bacterial infections in poultry, but small amounts can remain in their edible tissues. These residues may represent a risk for consumers due to allergic reactions and antimicrobial resistance. However, information on how long ampicillin remains in chicken tissues is still limited. Therefore, this study aimed to evaluate how long this drug remains in animal tissues and to define a safe waiting period before animals can be used for food. In this study, broiler chickens received intramuscular ampicillin, and muscle and skin samples were collected at different times after administration. Samples were analyzed using a sensitive laboratory method capable of detecting low levels of the antibiotic. Ampicillin was detected in muscle during the first day after administration only, while residues remained longer in skin but decreased over time. Based on these results, a waiting period of nine days was estimated to ensure that poultry meat is safe for human consumption, increasing to 19 days under a more conservative approach considering very low detectable levels of the antibiotic. These findings provide important information to support food safety evaluations and help authorities establish international safety limits for ampicillin in poultry, such as those developed by the Codex Alimentarius.

## 1. Introduction

Ampicillin is a broad-spectrum β-lactam antibiotic commonly used in poultry medicine to treat bacterial infections caused by *Escherichia coli, Pasteurella multocida,* and *Staphylococcus aureus*, and others [1]. It is especially effective in managing respiratory and systemic diseases, including those affecting the upper respiratory tract, lungs, and air sacs. As a valuable therapeutic tool, ampicillin helps to reduce morbidity and associated economic losses in farm animals [1,2].

Few pharmacokinetic studies have evaluated ampicillin in chickens. Ziv et al. [3] reported that a single dose of 25 mg kg^−1^ of ampicillin in chickens resulted in a maximum plasma concentration (C_max_) of 4.6 µg mL^−1^ at 0.5 h after intramuscular (IM) administration, with serum concentrations becoming undetectable by 6 h after treatment. In the same study, subcutaneous administration at 50 mg kg^−1^ produced a C_max_ of 4.2 µg mL^−1^ at 0.5 h, whereas oral bolus administration at 25 mg kg^−1^ resulted in a substantially lower C_max_ of 0.6 µg mL^−1^, also reached at approximately 0.5 h [3]. The authors indicated an elimination half-life of approximately 30 min after IM administration, though bioavailability for this route was not documented. Similarly, the study lacked specific half-life and bioavailability values for the SC and oral routes. More recently, Guzelaydin et al. [4] reported that oral administration of ampicillin at 25 mg kg^−1^ in chickens resulted in a C_max_ of 3 ± 0.47 µg mL^−1^, a time to peak concentration (T_max_) of 1.1 ± 0.31 h, and an oral bioavailability of 25.9%. The same authors reported a short elimination half-life for ampicillin, averaging 1.81 h after intravenous administration and 3.64 h after oral administration. However, plasma pharmacokinetics does not reflect residue depletion in edible tissues. Despite rapid elimination from plasma, ampicillin depletion may differ across tissues due to matrix-specific factors and physicochemical properties, influencing drug distribution and persistence [5].

Few studies have reported ampicillin residues in edible tissues of chicken. Ziv et al. [3] quantified 200 μg kg^−1^ of ampicillin in chicken muscle following a single oral bolus administration at 50 mg kg^−1^; however, edible tissues were only evaluated at a single early time point (4 h post-administration). Hamamoto and Mizuno [6] only evaluated samples collected two days after oral administration through feed, reporting higher concentrations in skin (6.30 ± 1.5 μg kg^−1^), while no detectable levels were found in the liver or kidney (<30 and 20 µg kg^−1^, respectively). Lakew et al. [7] also detected low ampicillin concentrations in chicken muscle (0.04 to 0.18 μg kg^−1^); however, their study lacked control over administration and sampling conditions, as samples were obtained from commercial sources. Similarly, monitoring studies in Vietnam [8] and Mexico [9] have reported the presence of β-lactam residues, including ampicillin, in poultry products from retail markets and commercial sources; however, they did not quantify residues.

More recently, Chughtai et al. [10] quantified ampicillin concentrations in muscle, liver, and kidney after broiler chickens received a commercially available ampicillin formulation for poultry by intramuscular injection into the pectoral muscle at 40 mg kg^−1^ body weight once daily for three consecutive days. The authors reported a decrease over time based on daily sampling during a 7-day post-treatment period, from approximately 2000 to 16 µg kg^−1^ in muscle, 1135 to 11.1 µg kg^−1^ in the liver, and 897.4 to 12.3 µg kg^−1^ in the kidney. This study provides one of the few datasets based on defined dosing regimens and multiple sampling time points post-treatment, enabling a more robust characterization of depletion kinetics. However, skin plus fat in natural proportions, a matrix usually consumed together with muscle [11], was not included. Overall, controlled residue depletion studies in edible tissues of broiler chickens following ampicillin administration remain limited.

Understanding ampicillin residue persistence in edible tissues is essential to ensure food safety, as residues in foods from treated animals can trigger several adverse effects in humans, including allergic reactions, diarrhea, skin rashes, and disruption of the intestinal microbiota by promoting selective pressure for resistant bacteria [12].

To protect animal-food consumers, national and international organizations have established maximum residue limits (MRLs), defined as the highest concentration of a drug or its metabolites that can legally be present in animal-origin foods without posing a risk to consumer health [13]. While MRLs for ampicillin have been established in certain regulatory frameworks, such as the European Union [14], for specific edible tissues, no MRL has been established for poultry by the Codex Alimentarius, creating uncertainty in regulatory frameworks that rely on Codex standards [15].

To address this gap, the Codex Alimentarius emphasizes the need for scientifically robust studies to characterize residue behavior of ampicillin in poultry, including the depletion of the marker residue (ampicillin) in edible tissues, supported by validated analytical methodologies according to international and current guidelines [16,17]. These data are evaluated by bodies such as the Joint FAO/WHO Expert Committee on Food Additives (JECFA) and provide the technical basis for establishing MRLs in broiler chicken tissues, which in turn support the determination of withdrawal periods for specific pharmaceutical formulations across different countries [12,16].

The limited available information on ampicillin residue behavior in broiler edible tissues highlights the need for controlled depletion studies, using known dosing regimens and precise sampling designs to allow reliable withdrawal period estimation. Despite recent controlled studies, relevant gaps persist, particularly regarding the inclusion of skin plus fat in natural proportions, as well as the comparative evaluation with muscle, both of which are matrices used for regulatory assessment of residues in poultry. Therefore, the aim of this study was to evaluate the depletion of ampicillin in skin plus fat and muscle of broiler chickens following IM administration, using an analytical method validated in accordance with international guidelines. The findings provide relevant data to support JECFA risk assessment and contribute to the establishment of MRLs for ampicillin in Codex Alimentarius, thereby supporting regulatory decision-making and the harmonization of food safety standards.

## 2. Materials and Methods

### 2.1. Chemicals, Reagents and Standards

All chemicals were HPLC-grade or analytical-grade, including water, acetonitrile, methanol, chloroform and hexane as extraction solvents; ammonium acetate, formic acid and ammonia solution (25%) for mobile phase and reconstitution solutions; and potassium dihydrogen phosphate (KH_2_PO_4_) and disodium hydrogen phosphate (Na_2_HPO_4_) for the preparation of phosphate buffer (pH 7.5). These were purchased from Merck (Darmstadt, Germany).

As standard, ampicillin (93% purity) was used, purchased from Sigma-Aldrich (Merck KGaA, Darmstadt, Germany). The internal standards were amoxicillin-d4 (AMOX-D4, 95% purity) for muscle matrix, sourced from Toronto Research Chemicals (Toronto, ON, Canada), and ampicillin-d5 (AMPI-D5, 95% purity) for skin plus fat matrix, purchased from Sigma-Aldrich (Merck KGaA, Darmstadt, Germany).

Stock solutions of each standard were prepared in methanol at a concentration of 1000 µg mL^−1^. From these, working solutions were subsequently prepared at 2.5 µg mL^−1^ in water:acetonitrile (1:1, *v*/*v*). One solution contained ampicillin, while a separate solution contained the internal standard mixture.

### 2.2. In-House Validation of Analytical Method

Before sample analysis of experimental birds, an in-house validation was performed following the protocol previously described by Pinto et al. [18], in accordance with the EU Commission Implementing Regulation (EU) 2021/808 [19] for quantitative confirmatory methods applied to veterinary drug residue analysis, VICH GL 2 [20], VICH GL 49 [21], and Sun et al. [22].

Method specificity was evaluated by analyzing 20 blank samples from each matrix, to ensure the absence of interferences at the retention time of ampicillin. The limit of detection (LOD) and limit of quantification (LOQ) were established using a minimum signal-to-noise ratio of 3:1 and 10:1, respectively. Linearity was assessed through matrix-matched calibration curves prepared at ascending and equidistant concentrations of 0, 25, 50, 75, 100, and 150 µg kg^−1^, with acceptance criteria of determination coefficient (R^2^) ≥ 0.95, and a *p*-value > 0.05 in the Mandel test.

Precision was determined at three levels (5, 50, and 75 µg kg^−1^), corresponding to 0.1, 1, and 1.5 times the European Union MRL for ampicillin. Repeatability was evaluated by the same operator on different days and within-laboratory reproducibility by different operators on separate days. These parameters were considered acceptable when the relative standard deviation (RSD%) did not exceed 16.6% (for 1 and 1.5 times) and 20% (for 0.1 times) under repeatability conditions, and 25% (for 1 and 1.5 times) and 30% (for 0.1 times) under within-laboratory reproducibility conditions.

Recovery was evaluated with acceptance limits of 70–120% for concentrations between 1 and 10 µg kg^−1^ and 80–120% for concentrations ≥ 10 µg kg^−1^. The decision limit (CCα) was calculated using the following Equation (1):

(1) CCα = MRL + 1.64 × SD

where *MRL* corresponds to the EU MRL for ampicillin (50 µg kg^−1^) and *SD* corresponds to the standard deviation of within-laboratory reproducibility for samples fortified at the MRL level. The final *CCα* value was expressed in µg kg^−1^, with α = 5%.

Method ruggedness was assessed by introducing variations in the extraction procedure, including the use of a non-adjusted buffer instead of a pH-regulated one, extending the final centrifugation time from 10 to 20 min, and evaporating at 45 °C instead of 30 °C under nitrogen flow. Standard deviation obtained under the modified conditions were considered indicative of method ruggedness when they were lower than the within-laboratory reproducibility standard deviation at the same fortification level (50 µg kg^−1^). The most critical factor was identified as the one showing the greatest standard deviation obtained between control and modified conditions.

Matrix effects were evaluated by fortifying the final extract of 20 blank samples with the working solution of ampicillin and internal standards, accepting an RSD ≤ 20%. Finally, stability was assessed by fortifying blank samples and analyzing them on the same day (time one), after 7 days (time two), and after 21 days (time three) post-fortification.

### 2.3. Animals and Housing

To determine ampicillin depletion in chicken tissues, 40 male broiler chickens from the Ross 308 genetic line were housed from day 1 of age in an Experimental Unit located at Faculty of Veterinary and Animal Sciences, University of Chile, which provided controlled conditions for temperature (25 ± 5 °C), humidity (50–70%), and ventilation in raised-floor cages, simulating commercial poultry production conditions. The facility included a clean area and an experimental area where the groups were housed. Birds had ad libitum access to water and non-medicated feed formulated according to breed-specific nutritional requirements. A poultry-specialized veterinarian supervised the health status and welfare of the animals throughout the study.

Animal management and slaughter procedures complied with Directive 2010/63/EU [23], and the slaughter of experimental birds complied with Regulation (EC) No. 1099/2009 [24]. The use of experimental animals and the protocol were approved by the Institutional Animal Care and Use Committee (CICUA) of the University of Chile (approval certificate No. 23643).

### 2.4. Depletion Study Design

Treated animals (Group A = 30 birds) were administered ampicillin intramuscularly in the pectoral muscle at 22 days of age (mean weight of 0.50 ± 0.08 kg), at a dose of 20 mg kg^−1^ every 24 h for three consecutive days, corresponding to a therapeutic dose according to Sumano and Gutiérrez [1]. The ampicillin formulation used was an injectable product intended for human use (500 mg powder vial, Vitalis^®^, Bogotá, Colombia), which was reconstituted with 5 mL of 0.9% sodium chloride solution to obtain a final concentration of 100 mg mL^−1^. This formulation was administered under extra-label conditions due to the absence of an authorized veterinary product for broiler chickens in the country.

Control animals (Group B = 10 birds) came from the same source and received the same management as Group A but were not treated. They were housed separately from treated animals in a different facility to avoid cross-contamination.

The study followed the European Medicines Agency guidelines: “Guideline on Determination of Withdrawal Periods for Edible Tissues” [25], and VICH GL48 “Studies to Evaluate the Metabolism and Residue Kinetics of Veterinary Drugs in Food-Producing Animals: Marker Residue Depletion Studies to Establish Product Withdrawal Periods” [26]. Six birds from Group A and two from Group B were slaughtered at each of the five sampling points: 12 h, day 1, 2, 5, and 9 post-administration. Both skin plus fat in natural proportions (from the pectoral and cervical regions, lower dorsal area, and legs) and the entire pectoral muscle were collected and stored at −20 °C.

### 2.5. Sample and Instrumental Analysis

Samples were subjected to solid–liquid extraction according to a previously implemented method [18]. Briefly, approximately 5 g of homogenized sample was fortified with internal standards (amoxicillin-d4 for muscle; ampicillin-d5 for skin plus fat) and extracted using phosphate buffer (pH 7.5) and chloroform. The extract was subjected to centrifugation and a hexane defatting step, followed by clean-up using conditioned Oasis HLB solid-phase extraction cartridges (6 cc vac cartridge, 200 mg sorbent per cartridge, 30 μm, 30/pk, from Waters™, Milford, CT, USA). After sample loading and washing with HPLC-grade water, analytes were eluted with acetonitrile. The eluate was evaporated under a nitrogen stream at 30 °C and reconstituted in an aqueous solution containing 0.01% formic acid and 0.2 mM ammonium acetate.

Then, a chromatographic analysis using LC–MS/MS was performed to detect and confirm ampicillin residues in each matrix. The system consisted of an Agilent 1290 Infinity series liquid chromatograph (Santa Clara, CA, USA) coupled to an AB Sciex API 5500 triple quadrupole tandem mass spectrometer (AB Sciex LLC, Framingham, MA, USA). The equipment was operated using Analyst 1.6.3 and MultiQuant 3.0 software. Chromatographic conditions and detector parameters were the same as described in Pinto et al. [18]. Specific mass conditions are detailed in Table 1.

Ampicillin concentrations in experimental samples were calculated using the regression equation obtained from matrix-matched calibration curves (R^2^ > 0.99), prepared at multiple concentration levels to prevent extrapolation from spiked blank samples of Group B (control birds). The linear regression equation was applied as follows:

(2) y = a + bx

where *y* represents the instrumental response (area ratio), *a* is the intercept, and *b* is the slope of the calibration curve. The analyte concentration (*x*) in the experimental samples was then calculated by rearranging Equation (2).

When concentrations were below the LOQ, values were replaced by one-half of the LOQ according to EMA guidelines [25]. Concentrations below the LOD were considered as not detected.

### 2.6. Depletion Time Estimation

To assess the depletion profile, the Guideline on the Determination of Withdrawal Periods for Edible Tissues [25,26] was followed.

Residue concentrations were naturally log-transformed, and a linear regression of the log-transformed concentrations versus time was performed. The statistical significance of the regression model was assessed based on the *p*-value of the slope (*p* < 0.05), and model assumptions were evaluated by testing the normality of residuals using the Shapiro–Wilk test (*p* ≥ 0.05) and homoscedasticity using Cochran’s test (*p* ≥ 0.05).

Withdrawal period was estimated based on the upper one-sided 95% confidence limit of the regression, defined as the time at which this limit intersects the decision limit. The European Union MRL for ampicillin (50 µg kg^−1^) and LOQ of the method (5 µg kg^−1^) were used as the decision limit. The resulting withdrawal periods were rounded up to the next whole day when decimal values were obtained.

## 3. Results

### 3.1. Analytical Method Performance and Validation

**Figure 1:**
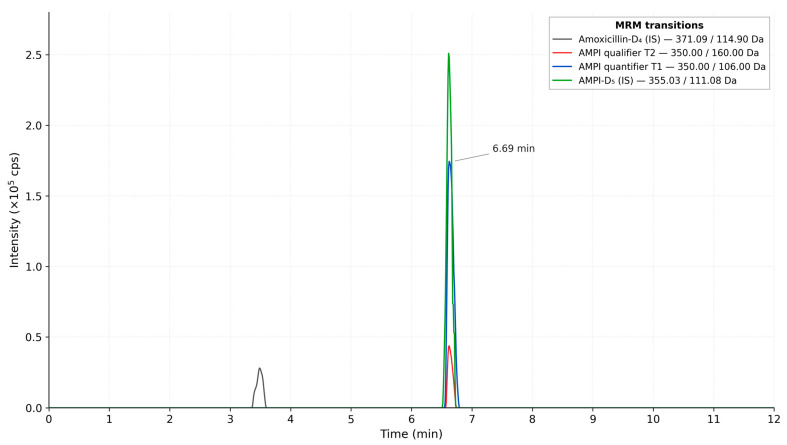
Representative chromatogram showing the signal intensities of the monitored ions under multiple reaction monitoring (MRM) conditions during liquid chromatography–tandem mass spectrometry (LC–MS/MS) analysis. The blue trace corresponds to ampicillin (AMPI) quantifier transition 1 (350.00/106.00 Da), the red trace to AMPI qualifier transition 2 (350.00/160.00 Da), the green trace to AMPI-D5 ion transition 1 (355.03/111.08 Da; internal standard), and the gray trace to amoxicillin-D4 ion transition 1 (371.09/114.90 Da; internal standard).

The analytes were detected using multiple reaction monitoring (MRM), as illustrated in the representative chromatogram shown in Figure 1.

The LOD and LOQ of the method were 3 and 5 µg kg^−1^, respectively. The method showed linearity (R^2^ ≥ 0.98) with a *p*-value > 0.05 in the Mandel test for all curves tested, indicating an adequate fit to the calibration model. Precision values ranged from 1.08% to 13.35% for repeatability and from 3.32% to 29.06% for within-laboratory reproducibility. Recovery values were close to 100%, ranging from 96.65% to 101.44%. The matrix effect was lower than 20% for both matrices. Finally, the decision limit (CCα) was lower than 57.50 µg kg^−1^. Further details are provided in Table 2.

The method showed adequate ruggedness in both matrices, with standard deviations of 0.16 µg kg^−1^ for skin plus fat and 0.04 µg kg^−1^ for muscle, lower than the corresponding within-laboratory reproducibility standard deviations of 3.86 and 4.56 µg kg^−1^, respectively. The most critical ruggedness factor was evaporation at 30 °C under a nitrogen stream for both matrices.

In general, the compound remained relatively stable in muscle with concentrations showing less than 5% variation between sampling times (Table 3). Conversely, a marked decrease was observed in skin plus fat, where ampicillin concentrations declined by approximately 90% after three weeks.

### 3.2. Ampicillin Quantification in Chicken Tissues

**Figure 2:**
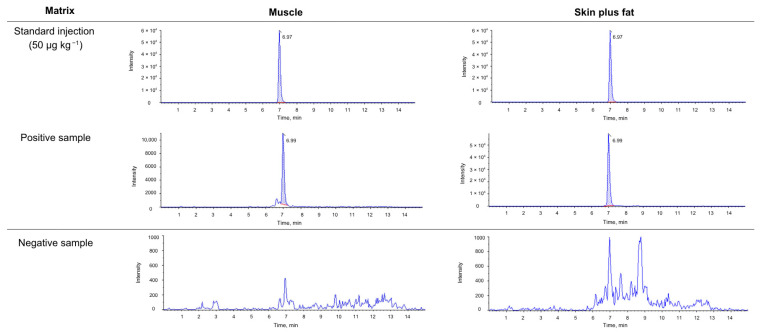
Chromatograms showing ampicillin detection in standard injection, positive samples from treated birds, and negative samples from the untreated control group.

Ampicillin was detected in both skin plus fat and muscle. Figure 2 shows chromatograms of positive samples compared with injections of pure standards and negative samples (the control group).

Detailed individual data on ampicillin residue quantification are presented in Table 4. In muscle, residues were detected only at the first two sampling times (12 h and 1 day post-administration), ranging from 6.50 to 8.48 µg kg^−1^, and thereafter were below the method LOD, so they were classified as not detected. In contrast, residues in skin plus fat persisted for a longer period, with measurable concentrations up to 5 days post-administration ranging from 59.88 to 6.87 µg kg^−1^. Although concentrations generally showed a decreasing trend over time, variability among sampling points was observed. No residues were detected in either matrix at day 9, except for a single bird showing 11.74 µg kg^−1^ in skin plus fat.

### 3.3. Ampicillin Residue Depletion in Chicken Matrices

In muscle, only two sampling points were quantifiable; therefore, no withdrawal period was calculated.

**Figure 3:**
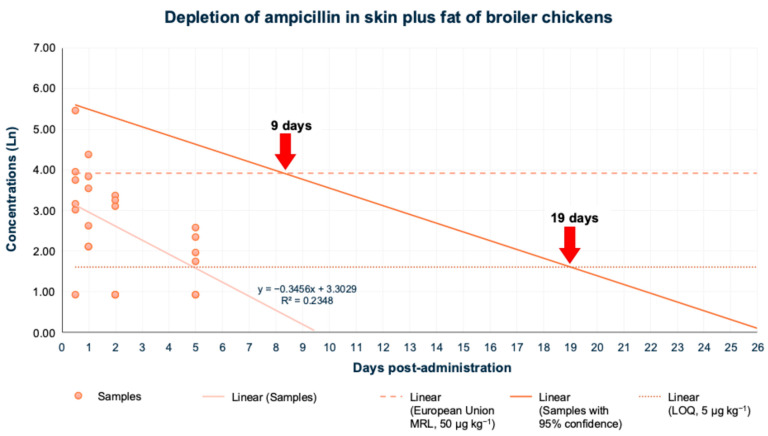
Regression analysis of ampicillin residue depletion in skin plus fat in natural proportions of broiler chickens following intramuscular administration. Log-transformed concentrations of the samples (circles; sampling points from 12 h to day 5) and the upper one-sided 95% confidence limit are plotted against days post-administration. The 9-day sampling point was excluded from the regression because most samples were not detected. By extrapolating the upper one-sided 95% confidence limit of the regression over time, the estimated withdrawal period for ampicillin in skin plus fat was 9 days when using the European Union maximum residue limit (MRL, 50 µg kg^−1^) as the decision limit. When the method limit of quantification (LOQ, 5 µg kg^−1^) was used as the decision limit, the estimated withdrawal period increased to 19 days. Red arrows indicate the estimated withdrawal periods obtained using each decision limit.

In skin plus fat in natural proportions, the linear regression model was statistically significant (*p* = 0.0152) with a slope of −0.3498, and the assumptions for linear regression were met under these conditions (Shapiro–Wilk: W = 0.9579, *p* = 0.3979; Cochran’s test: C = 0.4222, *p* = 0.4388). Based on this analysis, and using a 95% confidence level, a withdrawal period of 9 days was established using the EU MRL as the decision limit and 19 days using the method LOQ. The corresponding depletion profile is shown in Figure 3.

## 4. Discussion

The analytical method for ampicillin met all validation parameters of international guidelines, in both skin plus fat and muscle of broiler chicken, ensuring high specificity and sensitivity for the analysis of experimental samples. Matrix stability results showed that ampicillin degraded more rapidly in skin than in muscle, decreasing 89.67% compared to 3.46% in muscle over three weeks; therefore, it is important to process skin plus fat samples promptly after collection, as delays may lead to underestimation of residue concentrations. This instability is consistent with the known susceptibility of β-lactams to hydrolysis of the nucleophilic β-lactam ring under both acidic and basic conditions [27], a process further accelerated by tissue enzymes, pH, and water redistribution in biological matrices [28]. In this regard, Verdon et al. [29] reported significant degradation of incurred ampicillin residues in porcine muscle stored al −20 °C, while Freitas et al. [30] and Usanova et al. [28] demonstrated similar instability of amoxicillin and ampicillin residues, respectively, in chicken muscle under the same storage conditions, with all three studies consistently recommending ultra-low temperatures to preserve residue integrity. To our knowledge, no previous study has directly compared ampicillin stability between skin plus fat and muscle matrices; therefore, the greater degradation observed in skin plus fat in the present study represents a novel finding, which may reflect matrix-specific differences in water content, pH, and enzymatic activity [28] and warrants further investigation.

In this study, ampicillin residues reached higher concentrations in the skin (59.88 ± 87.07 µg kg^−1^) than in muscle (8.48 ± 8.71 µg kg^−1^), which may be attributed to the pharmacokinetic properties of ampicillin, including wide distribution, and relatively low plasma protein binding (17%) which increases the free fraction available for diffusion into peripheral tissues, allowing the drug to reach peripheral tissues such as skin [31,32,33].

A large proportion of ampicillin in skin is consistent with the findings of Hamamoto and Mizuno [6], who reported the presence of residues in skin and the absence of relevant concentrations in other edible tissues of broiler chickens two days post-administration. However, the mean concentrations reported by those authors (6.30 µg kg^−1^ in skin at day 2 post-administration) were nearly half of those observed in the present study (13.99 µg kg^−1^ at day 2). These differences may be attributed, at least in part, to variations in the route of administration, experimental conditions and pharmaceutical formulation. Oral administration via feed may result in lower and more variable systemic exposure due to incomplete gastrointestinal absorption and differences in individual feed intake, whereas IM administration provides a more direct and controlled delivery, ensuring that each animal receives the intended dose [5].

This pattern of greater residue accumulation in peripheral tissues has also been described in other studies of aminopenicillins in broiler chickens. For instance, Pinto et al. [18] observed a tendency for higher accumulation of amoxicillin residues in skin plus fat, reaching concentrations of 28.63–5.39 µg kg^−1^ within the first five days post-treatment, whereas no significant concentrations were detected in the muscle, liver, or kidney.

Lower concentrations detected in muscle compared to skin can be attributed to higher blood perfusion, which facilitates faster drug clearance. In contrast, the lower vascularization of skin and fat tissues may delay drug elimination, contributing to the prolonged persistence of ampicillin residues in skin plus fat [5,31]. This likely explains the absence of detectable residues in muscle samples as early as 48 h post-administration, while residues remained detectable in skin for a longer period.

However, some studies have reported higher and/or more prolonged persistence of ampicillin residues in muscle compared to the present findings. For example, Ziv et al. [3] detected concentrations of 200 µg kg^−1^ in muscle 4 h after a single high oral dose (50 mg kg^−1^), which may result in different pharmacokinetic behavior compared to the administration in the present study. Similarly, Chughtai et al. [10], reported detectable residues in muscle for up to seven days after IM administration, with concentrations decreasing from 1335.1 to 9.2 µg kg^−1^. These differences may be partially explained by variations in pharmaceutical formulation, as their study used an injectable suspension, whereas in the present work ampicillin was reconstituted in physiological solution. The higher viscosity of suspension formulations may delay absorption from the injection site, promoting prolonged persistence in muscle [5].

The withdrawal period estimated in the present study for ampicillin in edible tissues of broiler chickens (9 days) was longer than that reported by Chughtai et al. [10] (6 days), with both studies using the European Union MRL as the decision limit (50 mg kg^−1^). This difference may be attributed, firstly, to the fact that their study did not include the skin plus fat in natural proportion matrix, where the highest residue concentrations were observed in the present work. In addition, relevant methodological differences exist, as Chughtai et al. [10] proposed the withdrawal period based on the first sampling point where all matrices showed concentrations below the MRL. In contrast, this study followed EMA [25] guidelines, which recommend the use of statistical confidence for withdrawal period estimation. The withdrawal period was estimated using a log-linear regression approach, considering the upper one-sided 95% confidence limit, which allows the incorporation of biological and analytical variability of the experiment into the estimation and improves the statistical reliability of the withdrawal period estimation.

Furthermore, when the LOQ of the method was used in this study as the decision limit, a more conservative withdrawal period of 19 days was obtained. However, this value should not be interpreted as an alternative regulatory withdrawal period equivalent to the MRL-based estimate, since it is dependent on the analytical sensitivity of the method rather than on an established regulatory threshold. Instead, in this work represents a conservative analytical scenario that may provide additional information for risk-based assessments and residue monitoring strategies.

The use of ampicillin in this study was performed under extra-label conditions, as no authorized pharmaceutical formulation is available for broiler chickens in the country. The administration regimen was therefore based on literature data and reflects a plausible and controlled therapeutic scenario. Although regulatory frameworks establish that residue depletion and withdrawal periods are formulation-specific and must be defined under approved conditions of use [34], the present study provides relevant data on ampicillin depletion under controlled experimental conditions.

Besides the pharmaceutical formulation, variability in residue depletion may also arise from inter- and intra-species differences. Within poultry, factors such as genetic background, basal metabolic rate, body temperature, and glomerular filtration rate, as well as age, health status, and sex, may influence drug metabolism and excretion [5,35,36], thereby affecting the pharmacokinetics and persistence of antimicrobials in tissues. These sources of biological variability should be considered when interpreting residue depletion data and extrapolating withdrawal periods across different production settings.

In the present study, data variability is observed at some sampling points. This variability may be explained by inter-individual differences among birds, including differences in absorption from the intramuscular injection site, local behavior of the pharmaceutical formulation, tissue distribution, metabolism, and excretion [5,25,31]. Analytical uncertainty may also have influenced the dispersion of results, especially at low residue concentrations, where factors such as instrumental noise, signal interferences, and extraction or clean-up losses may become more relevant; this may be particularly important for ampicillin, considering its labile nature as a β-lactam molecule [37,38].

Among the study limitations is the inability to establish a withdrawal period in muscle, as fewer than three quantifiable sampling points were obtained, as required by the EMA guideline [25]. Further studies should increase the number of sampling points, particularly closer to the end of the treatment period, and assess the potential establishment of a zero-day tissue withdrawal period in accordance with VICH GL48. Another limitation is that only the marker residue of the antimicrobial (ampicillin) was evaluated. For studies involving antimicrobials intended for food-producing animals, it is also important to consider total residues, including metabolites such as ampicilloic acids, which, although not microbiologically active, may still trigger hypersensitivity reactions in consumers [39].

## 5. Conclusions

This study characterized the depletion of ampicillin residues in edible tissues of broiler chickens, using a validated quantitative–confirmatory analytical method in accordance with EU Regulation 2021/808. Under the specific formulation, dose regimen, intramuscular administration route, and experimental conditions evaluated in this study, the withdrawal time for skin plus fat was estimated at 9 days post-treatment. The results showed that residues persist longer in skin plus fat than in muscle, highlighting tissue-specific differences in residue depletion following IM administration.

Further studies should evaluate both total and marker residues of ampicillin in additional edible tissues, such as liver, gizzard, kidney, among others, to provide a more comprehensive understanding of residue behavior and to support robust risk assessments for regulatory decision-making in poultry production.

## Figures and Tables

**Table 1:** Mass spectrometric conditions for muscle and skin plus fat analysis in API 5500.

Analyte	Q1 Mass (Da)	Q2 Mass (Da)	Time (sec)	DP ^a^ (V)	EP ^b^ (V)	CE ^c^ (eV)	CXP ^d^ (V)
Ampicillin 1	350.00	106.00	150.00	50.00	7.00	25.00	10.00
Ampicillin 2	350.00	160.00	150.00	50.00	7.00	17.00	21.00
Ampicillin-d5 1	355.03	111.08	150.00	61.00	10.00	23.00	22.00
Ampicillin-d5 2	355.03	84.03	150.00	61.00	10.00	73.00	6.00
Amoxicillin-d4 1	371.09	114.90	150.00	66.00	10.00	23.00	55.00
Amoxicillin-d4 2	371.09	354.00	150.00	66.00	10.00	11.00	12.00

^a^ Declustering potential. ^b^ Entrance potential. ^c^ Collision energy. ^d^ Collision cell exit potential.

**Table 2:** Validation parameter results.

Matrix	Precision			Recovery (RSD %)	Linearity		CCα (µg kg^−1^)	Matrix Effect (%)
	Spike Level (µg kg^−1^)	RSD_WLr_ (%) ^a^	RSD_WLR_ (%) ^b^		Mean R^2 c^	*p*-Value ^d^		
Muscle	5	8.96	29.06	100.39	0.99	≥ 0.23	57.47	11.42
	50	2.52	9.43	96.65				
	75	1.08	3.85	101.44				
Skin plus fat in natural proportion	5	13.35	28.83	98.77	0.98	≥ 0.21	56.32	11.14
	50	3.68	7.62	100.34				
	75	1.58	3.32	99.85				

^a^ Relative standard deviation of repeatability data. ^b^ Relative standard deviation of within-laboratory reproducibility data. ^c^ Coefficient of determination. ^d^ Minimum Mandel test *p*-value obtained from three calibration curves.

**Table 3:** Stability of ampicillin mean results (*n* = 5).

Matrix	Time 1 ^a^	Time 2 ^b^	Time 3 ^c^	Change Time 2 vs. Time 1 (%)	Change Time 3 vs. Time 1 (%)
Muscle	51.13 ± 3.06	48.54 ± 3.35	49.36 ± 3.01	−5.07	−3.46
Skin plus fat in natural proportion	45.61 ± 1.92	23.98 ± 0.98	4.71 ± 0.267	−47.42	−89.67

^a^ Analysis performed on the same day of fortification. ^b^ Analysis performed one week after fortification. ^c^ Analysis performed three weeks after fortification.

**Table 4:** Ampicillin residue concentrations in broiler chicken pectoral muscle and skin plus fat in natural proportions at each sampling point following intramuscular administrations.

Sampling	Day of Life	Time Post-Administration	Bird ID	Skin Plus Fat		Muscle	
				Concentrations (µg kg^−1^)	Mean ± S.D. ^1^ (µg kg^−1^)	Concentrations (µg kg^−1^)	Mean ± S.D. (µg kg^−1^)
1	23	12 h	1.1	2.50 ^2^	59.88 ± 87.07	6.08	6.50 ± 2.72
			1.2	42.15		7.19	
			1.3	9.13		2.50 ^2^	
			1.4	233.37		6.13	
			1.5	51.72		6.13	
			1.6	20.40		10.99	
2	24	1 day	2.1	8.15	31.54 ± 27.96	5.13	8.48 ± 8.71
			2.2	13.70		5.86	
			2.3	79.35		6.10	
			2.4	34.14		26.06	
			2.5	45.76		5.26	
			2.6	8.14		2.50 ^2^	
3	25	2 days	3.1	28.64	13.99 ± 12.76	2.50 ^2^	- ^4^
			3.2	2.50 ^2^		ND ^3^	
			3.3	2.50 ^2^		ND	
			3.4	22.10		ND	
			3.5	25.72		ND	
			3.6	2.50 ^2^		ND	
4	28	5 days	4.1	5.64	6.87 ± 4.28	ND	-
			4.2	2.50 ^2^		ND	
			4.3	2.50 ^2^		ND	
			4.4	7.06		ND	
			4.5	13.13		ND	
			4.6	10.41		ND	
5	32	9 days	6.1	ND	-	ND	-
			6.2	11.74		ND	
			6.3	ND		ND	
			6.4	ND		ND	
			6.5	ND		ND	
			6.6	ND		ND	

^1^ S.D.: standard deviation. ^2^ Values reported as 2.50 µg kg^−1^ correspond to concentrations detected between the limit of detection (LOD = 3 µg kg^−1^) and the limit of quantification (LOQ = 5 µg kg^−1^) of the method, and were replaced by one-half of the LOQ according to EMA guidelines [25]. ^3^ ND or not detected: below the method limit of detection (3 µg kg^−1^). ^4^ “-“: indicates that the mean concentration was not calculated because most samples were not detected.

## Data Availability

This article includes all data generated or analyzed during this study. Upon reasonable request, additional details may be obtained from the corresponding author.
